# Nutritional Management of Patients with Fatty Acid Oxidation Disorders

**DOI:** 10.3390/nu16162707

**Published:** 2024-08-14

**Authors:** Luis Peña-Quintana, Patricia Correcher-Medina

**Affiliations:** 1 Pediatric Gastroenterology and Nutrition Unit, Insular Materno-Infantil University Hospital Complex, Asociación Canaria de Investigación Pediátrica, Centro de Investigación Biomédica en Red de la Fisiopatología de la Obesidad y Nutrición ISCIII, University of Las Palmas de Gran Canaria, 35016 Las Palmas de Gran Canaria, Spain; 2 Asociación Española para el Estudio de los Errores Congénitos del Metabolismo (AECOM), 28221 Majadahonda, Spain; 3 Metabolic and Nutrition Unit, Hospital Universitari i Politècnic la Fe, 46026 Valencia, Spain

**Keywords:** fatty acid oxidation disorders, energy metabolism, inborn errors of metabolism, fatty acid oxidation, intrahepatic lipids, hypoglycaemia, myopathy, cardiomyopathy, rhabdomyolysis, triheptanoin

## Abstract

Treatment of fatty acid oxidation disorders is based on dietary, pharmacological and metabolic decompensation measures. It is essential to provide the patient with sufficient glucose to prevent lipolysis and to avoid the use of fatty acids as fuel as far as possible. Dietary management consists of preventing periods of fasting and restricting fat intake by increasing carbohydrate intake, while maintaining an adequate and uninterrupted caloric intake. In long-chain deficits, long-chain triglyceride restriction should be 10% of total energy, with linoleic acid and linolenic acid intake of 3–4% and 0.5–1% (5/1–10/1 ratio), with medium-chain triglyceride supplementation at 10–25% of total energy (total MCT+LCT ratio = 20–35%). Trihepatnoin is a new therapeutic option with a good safety and efficacy profile. Patients at risk of rhabdomyolysis should ingest MCT or carbohydrates or a combination of both 20 min before exercise. In medium- and short-chain deficits, dietary modifications are not advised (except during exacerbations), with MCT contraindicated and slow sugars recommended 20 min before any significant physical exertion. Parents should be alerted to the need to increase the amount and frequency of carbohydrate intake in stressful situations. The main measure in emergency hospital treatment is the administration of IV glucose. The use of carnitine remains controversial and new therapeutic options are under investigation.

## 1. Introduction

**Figure 1:**
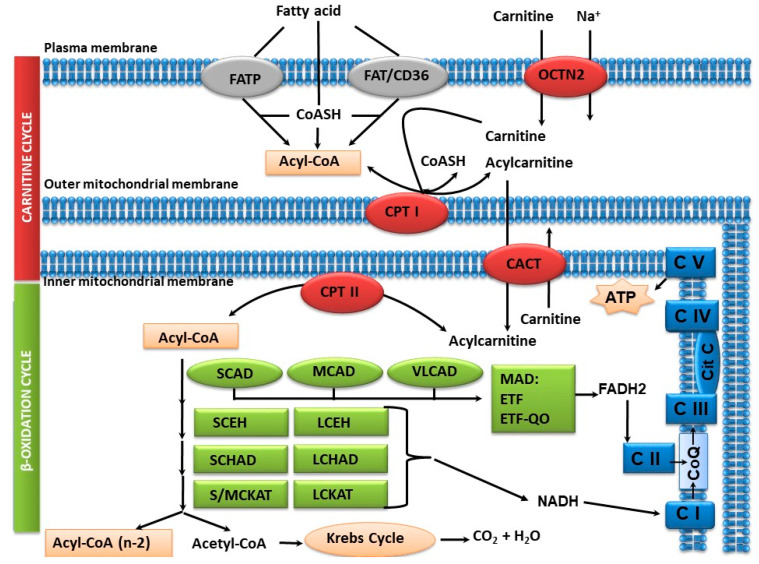
Carnitine and fatty acid ß-oxidation cycle. FATP, FAT/CD36: fatty acid transporters; OCTN2: carnitine transporter; CPT I: carnitine palmitoyltransferase I; CACT: carnitine acylcarnitine translocase; CPT II: carnitine palmitoyltransferase II; SCAD: short-chain acyl-CoA dehydrogenase; MCAD: medium-chain acyl-CoA dehydrogenase; VLCAD: very-long-chain acyl-CoA dehydrogenase; SCEH: short-chain enoyl-CoA hydratase; LCEH: long-chain enoyl-CoA hydratase; SCHAD: short-chain 3-hydroxyacyl-CoA dehydrogenase; LCHAD: long-chain 3-hydroxyacyl-CoA dehydrogenase; S/MCKAT: short/medium-chain 3-ketoacyl-CoA thiolase; LCKAT: long-chain 3-ketoacyl-CoA thiolase; MTP: mitochondrial trifunctional protein; MAD: multiple acyl-CoA dehydrogenase (CI, CII, CIII, CIV, CV) mitochondrial respiratory complexes I, II, III, IV and V.

Most tissues, except the brain, use fatty acid oxidation (FAO) for energy production through β-oxidation, which occurs in the mitochondria. Short- and medium-chain fatty acids can enter the mitochondria directly, while long-chain fatty acids require the intervention of three enzymes/transporters and carnitine. The heart, skeletal muscle and liver are particularly dependent on this pathway. Fatty acids are the preferred fuel of the heart (60–70% of energy) after birth and are also an important source of energy for skeletal muscle during prolonged exercise. The end product of FAO is acetyl-CoA, which can be utilised solely in the liver for the synthesis of ketone bodies that are driven to other tissues as auxiliary fuels such as the brain (which uses them as a major energy source during periods of prolonged fasting) or through the Krebs cycle and the mitochondrial respiratory chain to form CO_2_, H_2_O and ATP (Figure 1). On the other hand, in the liver, FAO provides energy for gluconeogenesis and ureagenesis.

FAO accounts for 80% of the body’s energy requirements in children after 12–24 h of fasting and in situations of metabolic stress (prolonged exercise, infections, fever, exposure to cold) in which the energy effect of glucose needs to be supplemented, once glycogen stores have been depleted, by mobilising fatty acids from adipose tissue. This process is of particular importance in the first days of life, where the foetal transition period occurs (where glucose is used as the main source of energy) to the neonate (where fat is the major energy source).

Fatty acid oxidation disorders (FAODs) are autosomal recessive inherited inborn errors of metabolism, including carnitine cycle disorders and defects of beta-oxidation in the mitochondrial matrix, involving at least 25 enzymes and transporters, with at least 22 associated genetic disorders (Figure 1) [1,2,3]. Due to the enzyme deficit, acetyl-CoA is not produced; gluconeogenesis, ureagenesis and ketone body formation are not activated, resulting in energy deficit, which can lead to hypoglycaemia without ketone body formation (except in short-chain and sometimes medium-chain ketones), lactic acidosis and hyperammonaemia.

After neonatal screening, the overall incidence is considered to be 1/9300 [2], although this varies between programmes and countries.

**Figure 2:**
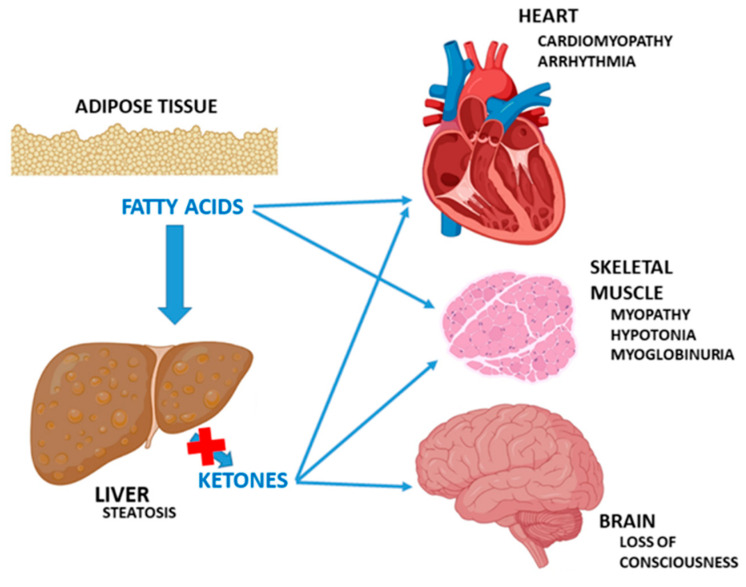
Defective fatty acid oxidation during fasting and metabolic stress. Adapted from Longo et al. [5,6].

The clinical spectrum and prognosis are highly variable depending on the enzyme deficit and the age of the patient, ranging from paucisymptomatic cases or mild symptoms in situations of metabolic stress, to more severe disease. Characteristically, FAODs in periods of fasting or metabolic decompensation present cardiac myopathy (colloquially referred to as “the machine without petrol”) or skeletal and/or hepatic involvement [4]. Neurological involvement may be mediated by the effects of hypoglycaemia, and by the toxic effects of the accumulation of fatty acids or their metabolites [5,6] (Figure 2). In inter-crisis periods, they are often found to be asymptomatic.

With the implementation of newborn screening, the clinical presentation and natural history has changed significantly. Diagnosis is mainly based on biochemical markers (acylcarntines, acylglycines, organic acids and other metabolites) and genetic testing [6].

## 2. Treatment of Fatty Acid Oxidation Disorders

Despite advances in the understanding of the pathophysiology of FAODs, therapeutic options are still limited and are still based on dietary, pharmacological and metabolic decompensation measures. It is essential to provide the patient with sufficient glucose to prevent lipolysis of adipose tissue, which is essential in the neonatal period and in metabolic decompensations.

### Dietary Treatment

The basis of dietary management is to prevent periods of fasting and to ensure sufficient calories during periods of metabolic stress, so as not to require the use of fatty acids as fuel as far as possible.

On the other hand, restriction of fat intake with increased carbohydrate intake, providing sufficient essential fatty acids, and maintaining an adequate and uninterrupted caloric intake are recommended [7,8].

To prevent periods of fasting, a healthy diet should be maintained using slowly absorbed carbohydrates with a low glycaemic index (Table 1) [4,9] to maintain adequate blood glucose levels:

- For this purpose, frequent meals throughout the day are advised, ensuring a constant supply of glucose. The optimal time between meals is not well established and may vary individually, depending on age, weight, growth and enzyme deficiency.
- Never skip breakfast.
- For children under 1 year, meals should be every 3–4 h.
- For children older than 1 year, meals can be every 4–5 h with never more than 8 h of fasting. The fasting periods are specified in Table 2 [8].
- It is advisable to have a midnight meal. Cornstarch can be used from 8 months of age when the pancreatic enzymes have the optimal capacity for adequate absorption. This preparation has a large amount of branched glucose chains that are hydrolysed and released slowly, allowing normoglycaemia figures to be maintained for 6–8 h, being more effective than an equivalent intake of glucose every 3 h. Start with 1.0–1.5 g/kg and gradually increase to 1.75–2 g/kg at 2 years of age. If other carbohydrate sources (rice, wheat, oats) are used, their effect may not be as desired due to lower amylopectin content [10].
- In severe cases with cardiomyopathy or feeding difficulties, nocturnal continuous enteral feeding by nasogastric tube or gastrostomy should be considered.

The dietary treatment is completed with a restriction of fat intake and an increase in carbohydrates. Quantitative fat restriction appears to be an appropriate measure, although the extent of fat restriction has not been agreed by consensus and whether it is necessary in all variants.

In childhood, total energy from fat should be between 40% and 45%, with a minimum of 10% long-chain fats and up to 20% in mild disease [11,12]. In childhood and adulthood, a restriction of 30–35% or less of fat from total calories is generally advised.

In patients with multiple dehydrogenase deficiency (MADD), in addition to fat restriction, a low-protein diet should be added with the aim of reducing excessive intake of isoleucine, leucine, lysine, tryptophan and valine [11].

Gillingahm MB et al. [13] propose that a modest increase in dietary protein in patients with long-chain deficits (LC-FAODs) could reduce hepatic fat without risk of metabolic decompensation, maintain lean body mass and metabolic control, and improve body composition and energy expenditure.

On the other hand, James P. DeLany et al. suggest that LC-FAOD patients have lower resting energy expenditure (REE) and therefore actually have slightly lower total energy expenditure (TEE) than estimated; current prediction equations may overestimate the energy expenditure of LC-FAOD patients [14].

Dietary management will be based on the following:

A. LC-FAODs (CPT-I, CPT-II, CACT, VLCAD, LCHAD/MTP):
  - Avoidance of fasting (primary measure) (midnight cornstarch or carbohydrates, nocturnal enteral nutrition).
  - Long-chain triglyceride (LCT) restriction to 10% of total energy (normalises plasma acylcarnitines), as restrictions below this figure entail a high risk of essential fatty acid deficiency and higher figures increase their potentially toxic metabolites [15,16]. It is very useful to provide the family with a traffic-light-type diet with recommended, limited or in moderation, and non-recommended foods (Table 3) [9].
  - Linoleic acid (C18:2n6) and linolenic acid (C18:3n3) intake of 3–4% and 0.5–1%, respectively, of total calories, with a 5/1–10/1 ratio, to avoid the risk of essential fatty acid deficiency. To this end, vegetable oils should be included in the diet as a source of essential fatty acid precursors, within 10% of LCT. Soybean, walnut, canola, flaxseed, wheat germ, sunflower or safflower oils are recommended.
  - In patients with MTP complex disorders including long-chain 3-hydroxy- acyl-CoA dehydrogenase (LCHAD) deficiency, there is no consensus on the intake of docoxahexanoic acid (DHA) (C22:6n3). DHA deficiency has been suggested to contribute to the pathogenesis of chorioretinitis, although supplementation does not appear to prevent retinal degeneration [17]. However, several groups [8,17,18,19] advise 65 mg/day in children weighing less than 20 kg, 130 mg/day in children weighing more than 20 kg and 100–200 mg/day in adults. Other groups do not routinely prescribe it, adding walnut oil to the diet [20].
  - Medium-chain triglyceride (MCT) supplementation at 10–25% of total energy. The minimum amount to be administered is 10%, as lower amounts do not decrease abnormal metabolites [15,16]. After ingestion, MCT diffuses easily and directly into the venous system and consequently into the tissues, not requiring the carnitine system or long-chain enzymes for its metabolisation in which four molecules of acetyl CoA are produced. These oils contain a mixture of even-chain fatty acids of 8 (octanoate) (mainly), 10 (decanoate) and some 12 (dodecanoate) carbons in length, which vary according to presentation. It has been suggested that the best octanoate/decanoate ratio is 1:3 [16]. MCT suppresses the long-chain FAO, preventing the accumulation of toxic metabolites (long-chain 3-hydroxy fatty acid intermediates), lactate and acylcarnitines [16]. This supplementation can be carried out in several ways.

○ Pure form at doses of 2–3 g/kg/day in the first year of life and 1–1.25 g/kg/day in those older than 1 year.
○ Complete diets with a high MCT content.
○ Modular, virtually fat-free diets such as those with the addition of LCT and MCT.
  - Triheptanoin (TH) is a highly purified odd-chain synthetic triglyceride composed of three seven-carbon fatty acids esterified with a glycerol. TH is catabolised in the gut to triheptanoate, which diffuses across membranes to enter cells. Its oxidation leads to two molecules of acetyl CoA and one of propionyl CoA, which through methylmanolyl CoA is irreversibly converted into succinyl-CoA (intermediate of the Krebs cycle), increasing the pool of these (anaplerotic function). This anaplerotic function of TH is what theoretically gives it added value, together with the increase in the production of oxaloacetate necessary for neoglucogenesis (improving glucose production) and the formation of aspartate and citrate.
  - Since its first publication in 2002 by Charles R Roe et al. [21], it has been used on a compassionate basis or in clinical trials in both non-responders to MCT therapy and naïve patients with different LC-FAODs (CACT, CPT I, CPT II, MTP, LCHAD, VLCAD) [22,23,24,25]. Different doses (3–4 g/kg/day in children; 1 g/kg/day in adolescents and adults; 20% total energy; 25–35% total energy) have been used, describing improvements in quality of life, cardiac function, muscle function and exercise tolerance, with reductions in mortality, time to hospitalisation for crises, number of episodes of hypoglycaemia, cardiomyopathy and rhabdomyolysis. TH has been approved by the FDA and can be administered from the neonatal period, has a good safety profile and in some patients induces diarrhoea (which improves after fractionation of the dose), abdominal pain or vomiting.
  - Total MCT+LCT = 20–35% of total energy.
  - In patients with mild to moderate disease, breastfeeding is not contraindicated, but should be supplemented by MCT-added formulas on a case-by-case basis. In symptomatic infants, discontinuation of breastfeeding should be considered, due to its high fat content [7,12,26].
  - Physical exercise: Patients with exercise-induced muscle pain or weakness (rhabdomyolysis) benefit 20 min prior to exercise from MCT at a dose of 0.25–0.5 g/kg of ideal weight for height [8,27] or carbohydrate at a dose of 1 g/kg of ideal weight for height or a combination of both at a lower dose. Rest and rehydration periods should be taken if training is long [18].
  - Monitor plasma levels of essential fatty acids and fat-soluble vitamins for risk of deficiency. There may be biochemical deficiency without clinical manifestations.

B Medium- and Short-Chain Deficiencies:
  - Maintain regular meals, avoiding prolonged fasting especially in the first 6 months of life [18].
  - When age permits, introduce foods containing slow sugars [28].
  - At present, dietary modifications are not recommended (except during exacerbations), maintaining a normal lipid intake according to WHO recommendations (30–35% of the total caloric value) [28], limiting saturated fatty acids and prioritising extra virgin olive oil and fats from foods such as avocado, nuts, seeds, etc.
  - The infant can be breastfed or take the usual infant formulas, checking their possible coconut oil content.
  - MCT is contraindicated in medium- and short-chain disorders and in multiple dehydrogenase deficiency (MADD). Modular diets without MCT can be used, taking great care not to ingest medium-chain fatty acids in the normal diet (formulas with MCT or processed products with MCT—mainly coconut).
  - Physical exercise: Intake of slow sugars at a dose of 1 g/kg of ideal weight for height 20 min before any notable physical exertion beyond usual activities, because of the risk of rhabdomyolysis. Limiting physical activity and school sports is not advised [8,18,27].
  - Considering that most short-chain acyl-CoA dehydrogenase deficiency (SCADD) patients diagnosed by newborn screening remain asymptomatic, it is suggested that SCADD is more likely a biochemical entity without clinical correlate, and no treatment or chronic management is necessary.

## 3. Nutritional Measures in Special Situations

### 3.1. Preoperative

If the patient requires fasting periods for any procedure, including dental extractions, intravenous administration of 10% glucose solution is recommended before, during and after the procedure until feeding is tolerated [20]. Cases of death have been reported in patients with unrecognised FAODs who have undergone dental extractions and required fasting prior to surgery.

### 3.2. Home Measures for Possible Decompensation

In stressful situations (infections, fever, vomiting, food refusal, etc.) at home, prolonged fasting should be avoided and a correct caloric intake should be ensured. Parents should be alerted to the need to increase in quantity and frequency the intake of carbohydrates such as fruit, jams, pasta, rice, bread, cornstarch, or gofio, without delaying these measures. In case of digestive intolerance, offer the emergency preparation according to the enzyme deficit (Table 4). If, despite taking it in small, frequent amounts, it is not tolerated, the patient should go to hospital for intravenous treatment.

## 4. Emergency Hospital Treatment

If the patient presents with vomiting, lethargy or general malaise, immediately administer 200 mg/kg of glucose (2 mL/kg of 10% glucose or 1 mL/kg of 20% glucose) within a few minutes. Follow with a maintenance glucose drip to ensure adequate blood sugar levels (110–120 mg/dL; 5–6 mmol/L) and hydration of the child. The use of a central line is preferable.

Hydration is essential during episodes of rhabdomyolysis and myoglobinuria to prevent renal failure, with dialysis being necessary in some situations when renal failure is imminent.

Polymer solutions of 10–20% glucose can be administered via nasogastric tube, alone or with i.v. glucose perfusion if the patient is not presenting with vomiting or diarrhoea, although i.v. perfusions are used in daily practise.

Delays in emergency treatment may result in sudden death or permanent brain damage. If the patient is in a coma, resolution may be rapid, although it can sometimes last 2–4 h or even 1–2 days, depending on the severity.

Low doses of insulin (0.05–0.1 U/kg/h) may also be administered when high amounts of i.v. glucose are required. If metabolic acidosis with pH < 7.20 is present, it should be rapidly corrected with i.v. sodium bicarbonate.

Hyperlacticaemia or hyperammonaemia need not be treated initially, if both are mild or moderate. If ammonium levels are higher than 150 µmol/L, treatment should be given, and carbamylglutamate may be used at a dose of 100 mg/kg orally in the first dose, followed by 100–250 mg/kg/day in four doses until ammonium levels are corrected.

There is no consensus on the use of carnitine in acute decompensation, the current tendency being not to use it [8] due to the possibility of producing toxic acylcarnitines, which can cause sudden death.

In case of liver dysfunction and coagulopathy, vitamin K should be added.

It is advisable for patients to have an information document (emergency letter) addressed to all physicians, especially emergency physicians, to authorise preferential referral to the emergency department, describing the disease, the circumstances that generate a risk of decompensation, the clinical risks and the measures to be taken in the event of decompensation [28].

Existing emergency protocols that are readily accessible for free can be found at https://www.filiere-g2m.fr/urgences (French and English) and www.emergencyprotocol.net (glycogenoses and FAOs; several languages available) accessed on 30 July 2024.

## 5. Pharmacological Treatment

On an individual basis and depending on the enzymatic deficit, pharmacological treatment will consist of the following:

Carnitine: The use of this detoxifier is still controversial, due to the risk of production of long-chain acylcarnitines and their toxic intramitochondrial accumulation, which has been associated with ventricular fibrillation and rhabdomyolysis [1]. It is absolutely essential in primary carnitine uptake or transport disorders, where high doses (200–400 mg/kg/day) can rescue possible cardiomyopathy, while in patients diagnosed by neonatal screening, an initial dose of 150 mg/kg/day divided into three doses is suggested, and it is advisable to maintain free carnitine levels above 20 µM in plasma. It is also indicated in pregnant women with this pathology, as it is not teratogenic.

It may be counterproductive in acylcarnitine transport disorders (CPT I, CPT II and CACT) [4].

In patients with MCAD, it can be used during the acute phase of the disease or in decompensations (50–100 mg/kg/d), where it functions as a scavenger of toxic metabolites (octanoyl-carnitine); however, in the chronic phase of the disease, its usefulness is more questionable.

Its systematic use in the remaining entities is controversial, with insufficient evidence for its supplementation (Cochrane 2012) [29].

Carnitine metabolism through the gut microbiota produces trimethylamine, which is subsequently converted to N-oxide-trimethylamine (TMAO) in the liver, an independent and dose-dependent risk factor for cardiovascular disease. Very high levels of TMAO have been detected in mitochondrial diseases under treatment with carnitine [30].

The current trend is not to prescribe it, except in the entities described above. It should only be considered and with great caution if carnitine levels are low, with low doses of 20–50 mg/kg/day in four doses before meals. In adults, 150 mg/kg/day is recommended.

High doses of carnitine may increase intestinal motility with diarrhoea or intestinal discomfort and/or produce trimethylamine with fishy body odour.

The diet provides 75% of the daily carnitine requirement in a healthy population [31], which could play an important role, especially in asymptomatic patients.

There are no published or ongoing randomised controlled clinical trials on its use.

Riboflavin is indicated at doses of 100–300 mg/day in all patients with MADD, as some of them respond to this cofactor.

Polyvitamin, a polyvitamin and mineral complex containing all fat-soluble vitamins (A, D, E, K), should be supplemented because of the risk of deficiency after dietary treatment [15]. Other groups only recommend it in documented deficiencies [15].

Coenzyme Q10 (ubiquinone) can be used when concomitant primary or secondary mitochondrial involvement is suspected, especially in MADD.

Advancements continue in the realm of gene therapy for common LC-FAODs.

Other therapeutic possibilities, such as Bezafibrate, REN001, Resveratrol, D, L-3-hydroxybutyrate (D, L-3-HB), nutritional ketones and Glycerol phenylbutyrate, are under investigation [32,33,34,35,36].

In patients with severe disorders and/or frequent decompensation, consider the placement of central venous access.

Drugs to avoid:

1. Anaesthetics

In patients with LC-FAODs, avoid volatile anaesthetics and those containing high doses of long-chain fatty acids such as propofol and etomidate, as well as muscle relaxants. Midazolam, fentanyl, thiopental, sevoflurane and nitrous oxide may be used as anaesthetics. In addition, propofol has been used in patients with LCHAD for short-term interventions without causing adverse events.

2 Other drugs to avoid:
  - Pivalic acid, valproic acid, salicylates and acetaminophen due to carnitine consumption.
  - Mildronate for decreasing carnitine synthesis and competing with the carnitine transporter (OCTN2).
  - Omeprazole, levofloxacin and various antitumour drugs (etoposide, vinblastine, actinomycin D) due to inhibition of OCTN2.
  - Statins for increasing OAG.
  - Adrenaline for its lipolytic effect [37].

## 6. Conclusions

In summary, the treatment of fatty acid oxidation disorders is based on dietary, pharmacological and metabolic decompensation measures. The main measure is to prevent lipolysis so as not to require the use of fatty acids as fuel as much as possible, while maintaining an adequate and uninterrupted caloric intake. For patients with long-chain deficiency, trihepatnoin is a new therapeutic option with a good safety and efficacy profile. Parents should be alerted to the need to increase the amount and frequency of carbohydrate intake in stressful situations. In emergency hospital treatment, the main measure is the administration of IV glucose. The use of carnitine remains controversial and new therapeutic options are under investigation.

## Figures and Tables

**Table 1:** Recommendations for maintaining a low-glycaemic-index diet [9].

▪ Prioritise children’s cereals without simple sugars. ▪ Promote the consumption of cereals such as oats and rye, or other cereals that are always wholemeal, as well as their flours. ▪ Ensure the consumption of pulses 3–4 times a week for their high fibre content. ▪ Consume vegetables and fruit daily, preferably with skin. Eat whole fruit, accompanied by other foods, and limit all types of juice. ▪ Encourage the retrogradation of starch in potatoes, pasta and rice by cooking them al dente and allowing them to cool (they can be reheated at the time of consumption). ▪ It is recommended to prepare menus based on the healthy plate: half a plate of vegetables, ¼ of a plate of age-appropriate protein foods and the other ¼ of wholegrain cereals.

**Table 2:** Fasting periods in stable metabolic state [8].

Age	Overnight Fasting Hours
Newborns	3
<6 months	4
6–12 months	6–8
>1 year	8–10

**Table 3:** List of foods according to their fat content in disorders of long-chain fatty acid oxidation (LC-FAODs) [9].

Foods	Advised Every Day (<1.5 g/100 g)	Limited Prescribed Amount (1.5–3 g/100 g)	Not Recommended On Prescription Only (>3 g/100 g)
Cereals and flour	Rice, pasta, flour, semolina, tapioca, barley flour, corn, others White loaf bread Cereal porridge-type cereals Corn, wheat or rice breakfast cereals	Quinoa, barley Italian pasta with egg Sliced bread Wholemeal bread Cereals with fibre Brown rice	Bread rusks Low-fat biscuits Oatmeal, bran and germ cereals Viennese pastries, croissants, biscuits, doughnuts, etc. Muesli, breakfast cereals with nuts, fillings, chocolate chips, etc.
Milk and dairy products	Skimmed milk and yoghurts Fresh cheeses and spreads, 0% fat	Milkshakes with semi-skimmed milk Semi-skimmed milk	Whole milk, plain, powdered or condensed milk Whole yoghurts Milk shakes with whole milk Cream Cheeses Cream ice creams
Fish, shellfish and molluscs	White fish such as haddock, cod, hake, megrim, sole, monkfish, whiting, others. Cockles, clams, squid, cuttlefish, octopus, Norway lobster, prawns. Yellowfin tuna in its natural state with less than 1g of fat x100 tuna.	Oily fish: anchovies, canned tuna Semi-fatty fish: Sea bream, sea bass, turbot Shellfish (mussels, red shrimp)	Oily fish: salmon, red mullet, sardines, halibut, trout Fried, pre-cooked fish Fish roe Fried squid
Meat and poultry	Skinless chicken and turkey breast. Rabbit Cooked low-fat ham. Turkey breast (cold meat).	Lean cuts of horse, pork and veal without visible fat Cooked and serrano ham Sausage loin	Pork, veal Fatty sausages, pâtés, sausages, offal.
Eggs	Egg white	-	Whole egg
Vegetables and root vegetables	All fresh and frozen	-	Pre-cooked or fried
Fruits and nuts	All fresh, except avocado and olives	-	Avocado, olives All nuts and dried fruits
Legumes	Lentils, beans, peas, broad beans	Chickpeas	Soya beans Soy bean and tofu drink
Oils and fats	MCT oil	On medical prescription	Olive/sunflower/soybean/nut oil, butter, lard, palm and coconut oil, margarines, mayonnaise
Sweets and desserts	Homemade jams and pastries made with skimmed milk and egg whites	Egg-free vanilla custard	Chocolates, soluble cocoa, marzipan, nougat Desserts made with whole milk, egg, cream Egg custard
Spices and sauces	All kinds of herbs, salt, lemon, homemade vegetable stock. Bechamel sauce with skimmed milk	-	Sauces made with cream, mayonnaise, butter, etc. Bechamel sauce with whole milk

**Table 4:** Emergency regime [9,11].

Disorders of Medium-Chain Fatty Acid Oxidation (MCAD), and Multiple Dehydrogenase Deficiency (MADD).					Disorders of Long-Chain Fatty Acid Oxidation (LC-FAODs) (CPT1, CPT2, CACT, LCHAD/MTP, VLCAD)		
Dextrinomaltose (DTM)					Medium-Chain Triglycerides (MCTs) Added to Dextrinomaltose		
Age (Years)	DTM (g/100 mL water)	Kcal/mL	Daily Volume	Calories/Day	MCT (mL/100 mL)	Kcal/mL (DTM + MCT)	Calories/Day (DTM + MCT)
0–1	10	0.4	150–200 mL/kg	60–80 Kcal/Kg	2	0.57	85–114 Kcal/g
1–2	15	0.6	100 mL/kg	60 Kcal/Kg	2	0.77	77 Kcal/Kg
2–6	20	0.8	1200–1500 mL	960–1200 Kcal	2	0.97	1165–1455 Kcal/Kg
6–10	20	0.8	1500–2000 mL	1200–1600 Kcal	2	0.97	1455–1940 Kcal/Kg
>10	25	1	2000 mL	2000 Kcal	2	1.17	2340 Kcal

The volume will generally be this figure divided by 12 and given 2-hourly during the day or night. If tolerance is good, it can be divided into 8 doses and given every 3 h.
